# Anaphylactoid Purpura Associated with Streptococcal Cellulitis: A Case Report and Literature Review

**DOI:** 10.1155/2017/5960898

**Published:** 2017-08-16

**Authors:** Yuko Saito, Susumu Ookawara, Hisataka Uchima, Takeshi Ishida, Masafumi Kakei, Hitoshi Sugawara

**Affiliations:** ^1^Division of General Medicine, First Department of Integrated Medicine, Saitama Medical Center, Jichi Medical University, 1-847 Amanuma-cho, Omiya-ku, Saitama-City, Saitama-ken 330-8503, Japan; ^2^Division of Nephrology, First Department of Integrated Medicine, Saitama Medical Center, Jichi Medical University, 1-847 Amanuma-cho, Omiya-ku, Saitama-City, Saitama-ken 330-8503, Japan; ^3^Department of Pathology, Saitama Citizens Medical Center, 299-1 Shimane, Nishi-ku, Saitama-City, Saitama-ken 331-0054, Japan; ^4^Department of Internal Medicine, Saitama Citizens Medical Center, 299-1 Shimane, Nishi-ku, Saitama-City, Saitama-ken 331-0054, Japan

## Abstract

A 54-year-old Japanese man noticed painful swelling and redness of his left leg. He was admitted for treatment of cellulitis, which was accompanied with increased anti-streptolysin O and anti-streptokinase titers in his clinical course. After Piperacillin/Tazobactam administration, the skin lesion resolved. However, the patient then developed arthritis, palpable purpura, and intermittent abdominal pain, later found to be secondary to a severe duodenal ulcer. He was diagnosed with cellulitis-associated anaphylactoid purpura and was given prednisolone, which dramatically improved his symptoms. The anaphylactoid purpura was likely caused by* Streptococcus*-induced cellulitis, which was successfully treated with prednisolone. Association between these diseases is rare.

## 1. Introduction

Anaphylactoid purpura, also known as Henoch-Schönlein purpura or immunoglobulin A vasculitis, is a systemic vasculitis of the small vessels [1]. This disease is histopathologically characterized by deposition of immune complexes between antigens and immunoglobulin A, and perivascular leukocyte infiltration in the skin, joints, gastrointestinal system, and kidneys [2]. Although there is no identification of its etiology, anaphylactoid purpura has been reportedly linked to a wide array of pathogens, including infections (bacterial, viral, and parasitic), pharmacological agents, vaccinations, and malignancies [3]. With regard to infections, anaphylactoid purpura frequently develops after upper respiratory tract infections, including sinusitis and focal infections of the oral cavity [4–7]. Consequently, various infectious factors are associated with the development of anaphylactoid purpura; however, few reports have detailed the association between anaphylactoid purpura and cellulitis.

In this case report, we describe a patient diagnosed with* Streptococcus*-induced cellulitis and subsequent development of anaphylactoid purpura successfully treated with prednisolone. This case report is relevant for clinicians practicing in both inpatient and outpatient settings.

## 2. Case Report

**Figure 1:**
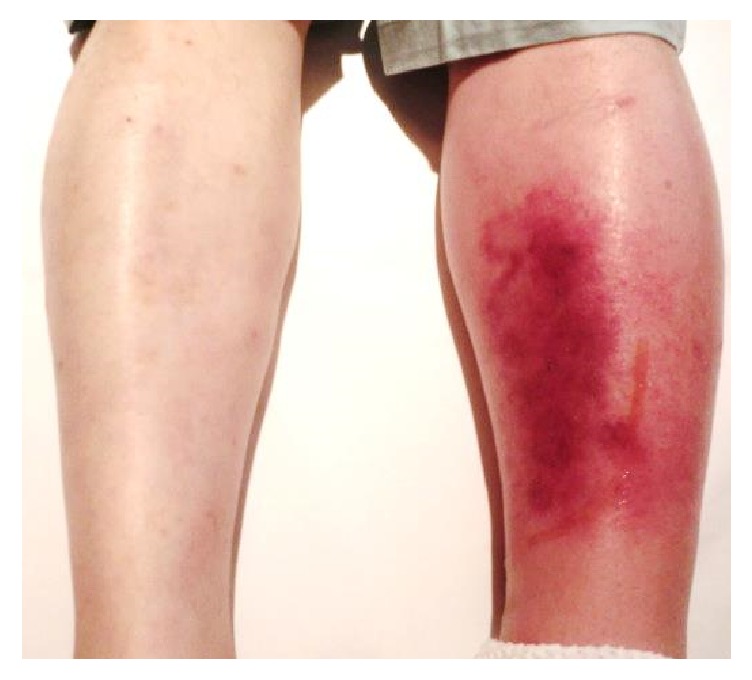
Photography of patient's cellulitis: swelling and redness of his left lower leg.

**Figure 2:**
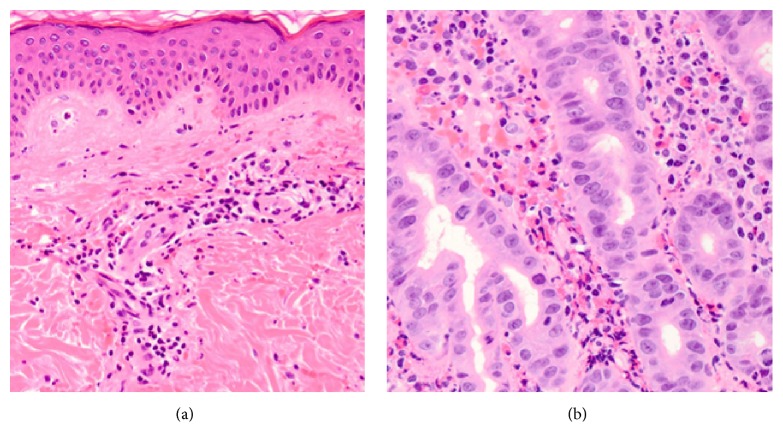
Histopathological findings. (a) Skin biopsy specimen shows neutrophil infiltrations around vessels in the upper dermis, also known as leukocytoclastic vasculitis (hematoxylin and eosin stain; original magnification ×100). (b) Duodenal biopsy specimen shows eosinophil infiltration of the mucous membrane (hematoxylin and eosin stain; original magnification ×400).

**Figure 3:**
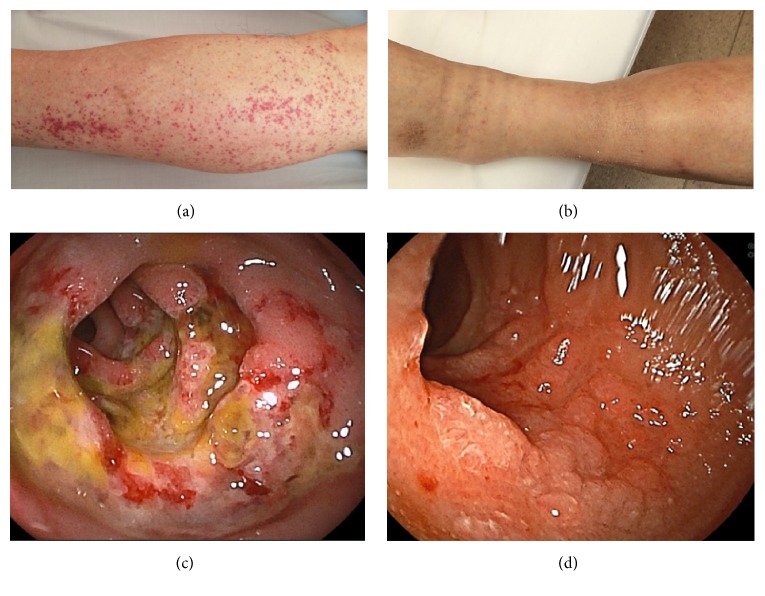
Changes of palpable purpura and duodenal ulcer before and after prednisolone administration. (a) Appearance of palpable purpura during the treatment of streptococcal cellulitis. (b) Improvement of palpable purpura after prednisolone administration for 2 weeks. (c) Severe duodenal ulcer with anaphylactoid purpura shown via endoscopy. (d) Improvement of duodenal ulcer after prednisolone administration for 2 weeks shown via endoscopy.

A 54-year-old Japanese man noticed painful swelling and redness of his left lower leg, which prompted him to visit a general practitioner. His symptoms were suspected to be due to cellulitis, and he was subsequently admitted to our hospital for the treatment of the cellulitis. His daily consumption of alcohol was 180 ml of whisky and his tobacco use amounted to 30 cigarettes per day. His past medical history included chronic hypertension and hyperuricemia. His medications included benzbromarone 25 mg/day, amlodipine 5 mg/day, and telmisartan 40 mg/day. On physical examination, a swollen, painful erythematous region was observed on his left lower leg. The area of erythema and swelling rapidly increased in size within a short period (Figure 1). Examination of the bilateral femoral lymph nodes was unremarkable, with no evidence of localized lymphadenitis in both sides. His height and weight were 171 cm and 77.0 kg, respectively; therefore, his body mass index was calculated as 26.3 kg/m^2^, which is classified as being overweight according to the World Health Organization criteria for adults [8]. His body temperature was 38.1°C, his heart rate was tachycardic (101 beats/minutes) with a regular rhythm, blood pressure was 124/78 mmHg, and oxygen saturation was 99% breathing room air. Laboratory data on admission showed an increased leukocyte count (13900/*μ*L) and elevated C-reactive protein level (9.48 mg/dL). A blood culture examination was repeated twice with both anaerobic and aerobic tubes; but all results were negative. Although the route of bacterial invasion to the skin was not determined, and there were neither known risk factors for infection nor any systemic immunodeficiency concerns other than an overweight and alcohol polydipsia [9], his cellulitis was suspected to be bacterial in origin due to its rapid progression within a short period. Therefore, Piperacillin/Tazobactam (4.5 g IV every 8 hours) was administered aiming to prevent further skin deterioration, septic shock, and the risk of amputation of his left lower leg. After 9 days of treatment administering Piperacillin/Tazobactam, the skin lesion resolved. However, during the evening of Day 9, the patient subsequently developed palpable purpura on the left lower leg. Furthermore, between Days 14 and 19, the patient reported symptoms of intermittent abdominal pain and painful joints swelling to right ankle, suggestive of acute ankle arthritis. Results of an esophagogastroduodenoscopy, performed on Day 34, revealed a severe ulcer with irregular and widespread lesions, located in the region of the descending portion of the duodenum. A skin biopsy specimen from a purpuric lesion revealed leukocytoclastic vasculitis in the upper dermis (Figure 2(a)) and a duodenal biopsy specimen from the duodenal ulcer also showed eosinophil infiltration into the mucous membrane (Figure 2(b)). Based on these clinical events despite no previous history of upper respiratory tract infections or any deterioration in renal function including urinalysis, the patient was diagnosed with anaphylactoid purpura, which was associated with bacterial cellulitis. As such, a daily oral administration of 40 mg prednisolone was initiated on Day 35 of his hospital stay. Thereafter, his symptoms gradually resolved, and esophagogastroduodenoscopy on Day 48 showed a dramatic improvement of the duodenal ulcer (Figure 3). Despite negative blood culture results, the anti-streptolysin O (ASO) and anti-streptokinase (ASK) antibody titers were elevated at 336 IU/mL (normal range < 239 IU/mL) and 2560 (normal range < 2560) on Day 35, respectively. Therefore, it is likely that the anaphylactoid purpura in this case was associated with a streptococcal infection. This patient was discharged as fully recovered and has been followed up successfully on an outpatient basis since then.

## 3. Discussion

Anaphylactoid purpura is diagnosed according to the European League against Rheumatism/Pediatric Rheumatology International Trials Organization/Pediatric Rheumatology European Society (EULAR/PRINTO/PRES) criteria. These criteria include palpable purpura and at least one of the four following phenomena: abdominal pain, histopathologic evidence of leukocytoclastic vasculitis or proliferative glomerulonephritis with immunoglobulin A deposits, arthritis or arthralgia, and renal involvement [13]. The incidence of this disease in adults is reported as 0.8–1.8/100,000, which is relatively rare [3]. In our patient, the diagnosis of anaphylactoid purpura was determined clinically upon identification of palpable purpura, abdominal pain, and arthritis of the ankle, although histopathological findings of the skin and duodenal biopsy specimens could not confirm immunoglobulin A deposition because of technical problems.

Anaphylactoid purpura has been reportedly associated with bacterial and viral infections [3, 14]. In particular, upper respiratory tract infections precede the development of anaphylactoid purpura in approximately 30% to 50% of cases (4–6) and have been considered the most common cause in the pathogenesis of anaphylactoid purpura. These infections result in the formation of immunoglobulin A-containing immune complexes in the blood, which can then deposit in small vessels throughout the body, leading to systemic vasculitis [2]. We used the PubMed database for our literature search, with emphasis on publications prior to October 2016, in both the Japanese and English languages, using the Medical Subject Headings terms cellulitis and anaphylactoid purpura. Using these search criteria, only three reports were found, which discussed the association between these diseases. We then reviewed the literature for reports of cellulitis-associated anaphylactoid purpura [10–12] (Table 1). All reported cases, including our cases, as shown in Table 1, were from Japan, although why Japan is the only country to report these particular cases remains unclear. Beta-hemolytic streptococci and* Staphylococcus aureus* are most commonly implicated as the causative agents of cellulitis, although a number of other microorganisms can uncommonly result in cellulitis.* Streptococcus* species were detected in the culture from the exudative fluid in only one patient with cellulitis. However, increased titers of ASO and ASK antibodies were confirmed in 3 cases. It was previously reported that the elevated titers of ASO and ASK antibodies were considered evidence of a recent streptococcal infection [15, 16]. Therefore, it is important to pay attention to the relationship between cellulitis, including streptococcal infection, and the development of anaphylactoid purpura. Regarding symptoms related to cellulitis-associated anaphylactoid purpura, only two patients, including our patient, presented with abdominal pain. In particular, our patient demonstrated a severe duodenal ulcer confirmed by esophagogastroduodenoscopy and required transient total parenteral nutrition. Therefore, this can be considered a very rare case from the viewpoint of the severity of abdominal symptoms accompanied by an unusual manifestation even among rare cases of cellulitis-associated anaphylactoid purpura. As to the treatment of cellulitis-associated anaphylactoid purpura, two out of five patients showed clinical improvement with no specific treatment; however, these two patients presented with skin lesions only and no accompanying systemic symptoms. Prednisolone administration was needed in two patients: one showed recurrence, and the other, in our case, developed systemic manifestations including severe abdominal pain and arthritis. Thus, in the treatment of cellulitis-associated anaphylactoid purpura, patients who experience recurrence and/or systemic symptoms may require prednisolone during their clinical course. On the other hand, administration of Piperacillin/Tazobactam can be associated with the pathogenesis of anaphylactoid purpura and could have been a relevant factor in this case. This agent is well-known to be a broad-spectrum antibiotic widely used for skin and soft tissue infections, abdominal infections, respiratory infections, complicated urinary tract infections, and gynecological infections. It is effective mainly against methicillin-sensitive coagulase-negative Staphylococci,* Streptococcus* pyogenes, penicillin-sensitive* Streptococcus pneumoniae*,* Haemophilus influenza*,* Pseudomonas aeruginosa*, and anaerobes [17]. Piperacillin/Tazobactam associated skin reaction, mainly petechial rash, was previously reported and disappeared completely within 3 days once this medication was discontinued [18]. In our case, palpable purpura progressively developed even after the discontinuation of Piperacillin/Tazobactam; therefore, it appears that there was limited association between anaphylactoid purpura pathogenesis and the administration of this agent in this case.

Pathological eosinophil infiltration was observed in the mucous membrane of the duodenal ulcer in this case. Recently, histopathologic results in 68 patients with Henoch-Schönlein purpura revealed eosinophil infiltration, in skin biopsy specimens in 51% of these patients, and also an inverse association between the presence of eosinophils and renal involvement [19]. Thus, the reason that renal involvement was absent in our patient's clinical course might be associated with the presence of eosinophils in the duodenal lesion, although the mechanism of eosinophil infiltration remains unclear.

In conclusion, we described a case of streptococcal cellulitis-associated anaphylactoid purpura. Based on our experience, we suggest that not only do patients with cellulitis need to be examined and treated using antibiotic agents, but additionally attention needs to be paid to the development of anaphylactoid purpura during their clinical course.

## Figures and Tables

**Table 1:** Case reports of cellulitis-associated anaphylactoid purpura.

Age	Sex	Location of cellulitis	AP occurrence from cellulitis (days)	Antibiotics for cellulitis treatment	Streptococcal detection by culture	ASO titer (IU/mL)	ASK (times)	Abdominal symptom	Treatment for AP	Reference number
78	M	Right lower leg	6	FOM	Negative (C)	287	2560	(+)	No treatment	[10]
50	M	Left lower leg	7	CEZ	Positive (C)	1610	640	(−)	No treatment	[11]
41	M	Left lower leg	3	CEZ	Negative (C)	ND	ND	(−)	DDS	[11]
78	F	Both lower legs	14	CEZ→SBT/ABPC	ND	ND	ND	(−)	PSL	[12]
54	M	Left lower leg	9	TAZ/PIPC	Negative (B)	336	2560	(+)	PSL	Current case

AP: anaphylactoid purpura, FOM: fosfomycin, CEZ: cefazolin, SBT/ABPC: sulbactam/ampicillin, TAZ/PIPC: tazobactam/piperacillin, (C): culture from exudative fluid of cellulitis, (B): blood culture, ND: no description, ASO: anti-streptolysin O antibody, ASK: anti-streptokinase antibody, DDS: diaminodiphenyl sulfone, and PSL: prednisolone.
